# The association between serum uric acid and cognitive performance in patients with ischemic stroke is modified by estimated glomerular filtration rate

**DOI:** 10.1038/s41598-023-34352-z

**Published:** 2023-05-02

**Authors:** Chunyan Zhang, Xiuping Zhang, Pengfei Meng, Huizhong Gao, Bo Bai, Dongfang Li

**Affiliations:** Department of Neurology, Second Hospital, Shanxi Medical University, 382 Wuyi Road, Taiyuan, 030001 Shanxi China

**Keywords:** Neuroscience, Nephrology, Neurology, Risk factors

## Abstract

The relationship between serum uric acid (SUA) and poor cognitive performance in patients with ischemic stroke is unclear. We hypothesized that the severity of renal function mediates the association between SUA and cognitive dysfunction.A retrospective analysis of 608 patients with ischemic stroke was conducted between 2016 and 2020. SUA was obtained from inpatient medical records. Global cognitive function via mini-mental state exam (MMSE) and Montreal Cognitive Assessment (MoCA) was determined one month after hospital discharge. The relationship between SUA and cognitive function was assessed by multiple linear and logistic regression analyses. Patients had a mean age of 66.6 years (SD: 4.1 years), and 52% were male. The mean SUA level was 298.6 ± 75.4 μmol/L. SUA increases were significantly positively associated with lower MMSE and MoCA scores and increased risk of moderate-severe cognitive impairment one month after stroke (*p* < 0.01), even after adjusting for factors including age, gender, BMI, diabetes and hypertension history. Adding a term for estimated glomerular filtration rate (eGFR) attenuated these associations such that SUA was no longer associated with cognitive performance. A fully adjusted stronger negative association between SUA and cognitive performance was found in those who had lower eGFR, with a significant eGFR interaction for MMSE (*p*-interaction = 0.016) and MoCA (*p*-interaction = 0.005). In patients with ischemic stroke, SUA showed an inverse association with cognitive function among those who have lower eGFR. The renal function might mediate the association between SUA and cognitive dysfunction.

## Introduction

Post-stroke cognitive impairment and dementia is a major source of morbidity and mortality worldwide^1^. A Chinese community-based study showed that the overall prevalence of post-stroke cognitive impairment (PSCI) is up to 80.97%, including 48.91% for cognitive impairment without dementia and 32.05% for dementia^2^. A better understanding of modifiable risk factors of cognitive decline or dementia after stroke is of vital importance to develop preventive strategies^3^. Serum uric acid (SUA) is the final product of purine catabolism and is excreted by the kidneys. Increased SUA is associated with a higher risk of vascular disease including stroke and chronic kidney disease (CKD)^4–6^, which may predispose individuals to cognitive impairment^7–9^.

To date, many cross-sectional or longitudinal studies have been performed to determine the relationship between uric acid and cognitive impairment, but the available data remain controversial and the link with renal function is not clear^10–13^. Recently, a meta-analysis found that a higher level of SUA after disease onset may be a marker of PSCI risk in patients with acute ischemic stroke^14^. A prospective study in patients with ischemic stroke and transient ischemic attack (TIA) in China found a U-shaped association between SUA and PSCI in males, but no association in females^15^. A cohort study in China found a negative correlation between SUA levels and MoCA scores in patients with lacunar cerebral infarction^8^. One cross-sectional study in patients with chronic heart failure demonstrated that elevated SUA is independently related to worse cognitive function in men but not in women^16^. One study in Turkey found uric acid levels are independently and inversely associated with mild cognitive dysfunction in subjects with CKD^17^. In contrast, a large population-based cohort did not find a relationship between SUA, eGFR (calculated using creatinine or cystatin C) levels and cognitive performance^18^. In a longitudinal study, hyperuricemia was associated with lower baseline mini-mental state exam (MMSE) scores but not with MMSE change over time, and also that albuminuria is an independent predictor of subsequent cognitive decline in men^19^.

SUA is commonly elevated in those with CKD and is associated with an increased risk of CKD progression^20^. A meta-analysis reported that uric acid lowering therapy may be effective in retarding the progression of CKD^21^. Furthermore, one study suggests that lowering uric acid in subjects with CKD may slow down the progression of renal disease and cardiovascular risk^22^. Similarly, early reduction in SUA may predict future improvement of renal function, which greatly positive affects the risk of cardiovascular events^23^ and cognitive dysfunction^24^.

The exact links between SUA and cognitive dysfunction in stroke patients are not fully understood. The main pathological mechanisms involved in SUA related cognitive dysfunction, such as oxidative stress, neuroinflammation, endothelial dysfunction and excitotoxicity, may collectively affect neuronal and brain function and further implicate SUA-related cognitive decline. SUA metabolism may be a double-edged sword in terms of the inflammatory and/or oxidative responses it induces in brain tissue, although its harmful effects appear to outweigh the benefits of SUA in most cases^25–27^. SUA as the compound of uremic toxicity is more likely to affect cerebro-renal interaction dysfunction, which may be the cause of cognitive impairment with CKD^28^. It is important to clarify the relationship between SUA and PSCI and whether this relationship is modified by renal function. This study aimed to investigate whether SUA is independently associated with worse cognitive performance in patients with ischemic stroke, and the influence of renal function.

## Methods

### Data source

Using a retrospective design, this study analyzed data from 608 patients aged 60 to 80 years with acute ischemic stroke within 72 h of onset between January 2016 and December 2020. Data were obtained from inpatient medical records and a database of cognitive assessment results for outpatient visits one month after hospital discharge. All patients had no history of a severe cognitive disorder or hyperuricemia before the stroke and were able to complete the assessment. Patients were excluded if they had a condition preventing them from completing the examination, such as aphasia or a consciousness disorder.

The study was conducted at the Department of Neurology, Second Hospital of Shanxi Medical University, Taiyuan, a tertiary hospital in Shanxi, China. This study was approved by the Ethics Committee of the Second Hospital of Shanxi Medical University (No. 2019YX214). The research was completed in accordance with the Helsinki Declaration. Due to the retrospective nature of the study, informed consent of the patients was not required.

### Clinical and laboratory data

Clinical and laboratory information extracted from hospital records included systolic and diastolic blood pressure, self‐reported demographic characteristics (age, gender, height, weight, years of education, smoking and alcohol consumption status, and diabetes mellitus and hypertension history); and laboratory results obtained on admission. Fasting venous blood was collected for analysis. Biochemical measurements were measured using automatic clinical analyzers (Beckman Coulter) at the core lab of the Second Hospital, Shanxi Medical University, Taiyuan, China. SUA was assayed by an uricase-peroxidase method. Day-by-day variation had to be within 5%. Values were set in international units (µmol/L). The reference range for adult men was 208.3–428.4 µmol/L, whereas for women, this range was cited as 154.7–357.0 µmol/L. Serum glucose was determined by a hexokinase method. Fasting lipids and serum creatinine concentrations were determined by an enzymatic method. Serum folate and vitamin B12 were measured using a chemiluminescent immunoassay. Urine albumin and creatinine levels were determined using an automatic protein analyzer (Alere Afinion AS100) at the lab of nephrology department.

Additionally, data on neural function were taken via the National Institutes of Health Stroke Scale (NIHSS) score. All patients were diagnosed according to the TOAST classification as large artery atherosclerosis, cardioembolism, small artery occlusion, stroke of other determined cause, or stroke of undetermined cause in the medical records^29^. Body mass index (BMI) was calculated as weight in kilograms divided by height in meters squared. The eGFR was calculated using the CKD-EPI equation.

$${\text{eGFR}} = {141} \times {\text{min}}({\text{Scr}}/{^{\kappa}},{1})\alpha \times {\text{max}}({\text{Scr}}/{^{\kappa}},{1}) - {1}.{2}0{9} \times 0.{\text{993age}} \times {1}.0{18 }\left[ {{\text{if}}\;{\text{female}}} \right]$$

where serum creatinine (Scr) is expressed in mg/dl and age in years; $\kappa$ is 0.7 for females and 0.9 for males; $\alpha$ is − 0.329 for females and − 0.411 for males; min indicates the minimum of Scr/^κ^ or 1, and max indicates the maximum of Scr/^κ^ or 1^30^.

### Cognitive impairment testing

All cognitive function tests were conducted by two neuropsychological assessors trained in consistency using the MMSE and Montreal Cognitive Assessment (MoCA) one month after hospital discharge. The good reliability and validity of MoCA and MMSE were confirmed in China^31,32^. These two commonly-used cognitive screening tools have similar accuracy for detection of dementia/multidomain impairment^33^. Compared to the MMSE, the MoCA has a higher sensitivity and specificity for initial cognitive function screening^34,35^, and has been reported to be particularly useful to discern PSCI in patients whose cognitive deficits were undetectable with the MMSE. The uses of the MoCA and MMSE should be combined to optimize the PSCI screening^36,37^. An extra point was added to the MoCA test score for patients with < 12 years of formal education. The MMSE and MoCA scores ranged from 0 to 30, with higher scores indicating better cognitive function. The tests assess attention, concentration, orientation, language, the ability to follow simple verbal and written commands, and immediate- and short-term recall. According to a previous study and cognitive scores of the patients, moderate-severe cognitive impairment one month after stroke was defined as a MoCA score < 17 or MMSE < 20^37^.

### Statistical analysis

Retrospective data were analyzed. Patient characteristics were presented as mean ± SD for continuous variables, and categorical variables were reported as frequencies and percentages. Differences between groups for continuous data were tested by one-way ANOVA. Differences between groups for proportions were tested using Chi-square. Multiple linear regression and logistic regression were used to estimate the β and odds ratio (OR) of SUA level with cognitive function. SUA levels were divided into: 58.0–246.9, 247.0–287.9, 288.0–339.9, and 340.0–611.0 μmol/L according to SUA quartiles. Clinical and laboratory variables were analyzed according to SUA grade levels. Crude and adjusted ß and OR with 95% CIs of cognitive scores and moderate-severe cognitive impairment were calculated for quartiles of uric acid (with the lowest quartile as the reference) as well as continuously, per 20 unit increase in uric acid level. Then, we combined Q1–Q3 and Q4 categories for further analysis.

We constructed three models with progressively increased adjustments for confounding variables that could affect the association between uric acid level and cognitive function. The first was crude. Model 1 was additionally adjusted for age, gender, systolic blood pressure, BMI, education, diabetes and hypertension history, stroke subtypes, NIHSS score, smoking and alcohol drinking status, serum total cholesterol, triglyceride, vitamin B12, folate, fasting glucose, and UACR. Model 2 was further adjusted for estimated glomerular filtration rate. A two-tailed *p* < 0.05 was considered statistically significant. All analyses were performed using Empower® (www.empowerstats.com; X&Y solutions, Inc., Boston, MA) and R (http://www.R-project.org).

### Ethics approval and consent to participate

This retrospective research was completed in accordance with Helsinki Declaration. This study was approved by the Ethics Committee of the Second Hospital of Shanxi Medical University (NO. 2019YX214). Due to the retrospective nature of the study, informed consent of the patients was not required.

## Results

### Population characteristics

The demographic and clinical characteristics of all 608 patients grouped by SUA quartiles are shown in Table 1. Patients had a mean age of 66.6 years (SD: 4.1 years) and 52% were male. The mean SUA level was 298.6 ± 75.4 μmol/L. The ranges of SUA for quartiles 1–4 were 58.0–246.9, 247.0–287.9, 288.0–339.9, and 340.0–611.0 μmol/L, respectively. Patients in the higher SUA categories were more likely to be male and former or current smokers and drinkers. In addition, they were less likely to have diabetes, but more likely have a higher level of education and DBP, lower blood glucose and eGFR on admission, and lower MoCA scores one month post-stroke (Table 1).

**Table 1:** Demographic and clinical characteristics of patients stratified by SUA.

	Overall	Uric acid (µmol/L)				*P* value
		Q1(58.0–246.0)	Q2(247.0–287.0)	Q3(288.0–339.0)	Q4(340.0–611.0)	
	n = 608	n = 151	n = 150	n = 155	n = 152	
Age, y	66.6 ± 4.1	66.4 ± 4.1	66.8 ± 4.3	66.9 ± 4.0	66.2 ± 4.2	0.463
Male, n (%)	316 (52.0)	46 (30.5)	67 (44.7)	87 (56.1)	116 (76.3)	< 0.001
BMI,^a^ kg/m^2^	25.0 ± 3.6	24.6 ± 3.3	24.9 ± 3.6	24.7 ± 3.6	25.6 ± 3.7	0.054
Education (> 6 years), n (%)	200 (32.9)	28 (18.5)	36 (24.0)	59 (38.1)	77 (50.7)	< 0.001
Systolic blood pressure on admission, mmHg	171.9 ± 20.2	172.4 ± 19.4	171.4 ± 18.5	172.9 ± 21.9	170.8 ± 21.1	0.795
Diastolic blood pressure on admission, mmHg	92.9 ± 12.2	90.2 ± 12.1	93.0 ± 12.4	93.8 ± 12.7	94.6 ± 11.5	0.011
Smoking, n (%)						
Never	355 (58.5)	118 (78.1)	98 (65.8)	80 (51.6)	59 (38.8)	< 0.001
Former	64 (10.5)	9 (6.0)	12 (8.1)	19 (12.3)	24 (15.8)	
Current	188 (31.0)	24 (15.9)	39 (26.2)	56 (36.1)	69 (45.4)	
Alcohol drinking, n (%)						
Never	389 (64.0)	119 (78.8)	105 (70.0)	96 (61.9)	69 (45.4)	< 0.001
Former	52 (8.6)	11 (7.3)	11 (7.3)	13 (8.4)	17 (11.2)	
Current	167 (27.5)	21 (13.9)	34 (22.7)	46 (29.7)	66 (43.4)	
Stroke subtypes						0.728
Large artery	281 (46.2)	71 (47.0)	72 (48.0)	66 (42.6)	72 (47.4)	
Small vessel occlusion	309 (50.8)	72 (47.7%)	75 (50.0%)	83 (53.5%)	79 (52.0%)	
Cardioembolism	13 (2.1)	6 (4.0%)	2 (1.3%)	4 (2.6%)	1 (0.7%)	
Other determined etiology	3 (0.5)	1 (0.7%)	1 (0.7%)	1 (0.6%)	0 (0.0%)	
Undetermined etiology	2 (0.3)	1 (0.7%)	0 (0.0%)	1 (0.6%)	0 (0.0%)	
Diabetes mellitus,^b^ n (%)	50 (8.2)	21 (13.9)	13 (8.7)	10 (6.5)	6 (3.9)	0.013
Hypertension, n (%)	361 (59.4)	89 (58.9)	90 (60.0)	95 (61.3)	87 (57.2)	0.906
Laboratory results						
Total cholesterol, mmol/L	5.7 ± 1.2	5.7 ± 1.1	5.7 ± 1.1	5.6 ± 1.2	5.8 ± 1.2	0.393
Triglyceride, mmol/L	1.6 ± 0.9	1.5 ± 0.7	1.6 ± 0.8	1.7 ± 1.2	1.7 ± 1.0	0.088
Folate, ng/mL	7.5 ± 3.3	7.9 ± 3.7	7.6 ± 3.4	7.2 ± 3.1	7.1 ± 3.0	0.138
Vitamin B12, pmol/L	399.5 ± 165.5	413.3 ± 177.6	376.1 ± 110.0	395.1 ± 176.4	413.7 ± 185.0	0.157
Uric acid, µmol/L	298.6 ± 75.4	212.4 ± 28.9	267.2 ± 11.8	313.6 ± 14.8	399.8 ± 52.2	< 0.001
Fasting glucose, mmol/L	6.1 ± 1.8	6.5 ± 2.4	6.1 ± 1.7	6.0 ± 1.7	5.9 ± 1.2	0.019
Estimated glomerular filtration rate, ml/min/1.73m^2^	88.4 ± 12.9	92.5 ± 8.8	90.1 ± 9.9	88.3 ± 12.8	82.7 ± 16.6	< 0.001
UACR, mg/g	83.6 ± 314.2	90.7 ± 392.5	71.8 ± 133.0	92.4 ± 443.8	79.4 ± 159.1	0.933
Neural function assessment, mean (SD)						
MMSE score, mean ± SD (range 0–30)	21.4 ± 2.1	21.6 ± 1.8	21.6 ± 1.8	21.5 ± 2.1	21.1 ± 2.6	0.101
MoCA score, mean ± SD (range 0–30)	17.9 ± 2.3	18.2 ± 2.0	18.0 ± 2.1	18.1 ± 2.4	17.4 ± 2.7	0.017
NIHSS score, mean ± SD (range 0–42)	5.0 ± 2.2	5.3 ± 2.3	4.9 ± 2.3	4.9 ± 2.2	4.9 ± 2.1	0.393

*SUA* serum uric acid, *BMI* body mass index, *UACR* urine albumin creatinine ratio, *MMSE* Mini-Mental State Examination, *MoCA* Montreal Cognitive Assessment, *NIHSS* National Institutes of Health Stroke Scale.

Values are presented as mean ± SD for continuous variables and n (%) for categorical variables.

^a^BMI was calculated as weight in kilograms divided by height in meters squared.

^b^Diabetes mellitus was defined as self-reported physician diagnosed diabetes.

^C^Hypertension was defined as self-reported physician diagnosed hypertension.

Q1: quartile 1 (lowest quartile); Q2: quartile 2; Q3: quartile 3; Q4: quartile 4 (highest quartile).

### SUA levels and cognitive performance

Table 2 contains the results of unadjusted and adjusted models for continuous and categorical relationships between parameters of SUA and cognitive tests. When examined as a continuous variable (per 20-unit increase) in both crude and model 1, SUA increases were also significantly positively associated with lower MMSE and MoCA scores (*p* < 0.01). However, after eGFR was introduced into the model, the relationship between SUA and cognitive performance disappeared (*p* = 0.396 for MMSE and *p* = 0.122 for MoCA). In the category analysis, we found a significant association for patients with the highest SUA quartile compared to patients with the lowest quartile in crude and model 1: MMSE (ß, − 0.49; 95% CI − 0.96, − 0.02 in crude and ß, − 0.60; 95% CI − 1.11, − 0.10 in model 1); and MoCA (ß, − 0.78; 95% CI − 1.30, − 0.26 in crude and ß, − 0.82; 95% CI − 1.38, − 0.26 in model 1). The direction of the relationship between SUA and cognitive function changed once eGFR was introduced into the model (ß, 0.45; 95% CI − 0.03, 0.92 in Model 2 for MMSE and ß, 0.60; 95% CI 0.10, 1.09 in Model 2 for MoCA). Furthermore, when we combined the first three-quarters of SUA as the reference, the outcomes were similar with the fourth quartile of SUA versus the first three-quarters (Table 2).

**Table 2:** Linear regression analysis of the association between SUA levels and cognitive measures.

SUA level (µmol/L)	No. of patients (%)	Crude		Model 1		Model 2	
		ß (95%CI)	*P*	ß (95%CI)	*P*	ß (95%CI)	*P*
MMSE							
Q1 (58.0–246.0)	151(24.8)	0		0		0	
Q2 (247.0–287.0)	150(24.7)	0.02 (− 0.45, 0.50)	0.921	0.01 (− 0.46, 0.47)	0.977	0.28 (− 0.13, 0.69)	0.183
Q3 (288.0–339.0)	155(25.5)	− 0.03 (− 0.50, 0.43)	0.887	− 0.00 (− 0.48, 0.47)	0.984	0.43 (0.01, 0.85)	0.046
Q4 (340.0–611.0)	152(25.0)	− 0.49 (− 0.96, − 0.02)	0.041	− 0.60 (− 1.11, − 0.10)	0.02	0.45 (− 0.03, 0.92)	0.065
*P* for trend		− 0.15 (− 0.30, − 0.00)	0.045	− 0.18 (− 0.34, − 0.02)	0.029	0.15 (0.00, 0.30)	0.047
Q1–Q3 (58.0–339.0)	356(75.0)	0		0		0	
Q4 (340.0–611.0)	152(25.0)	− 0.49 (− 0.87, − 0.10)	0.013	− 0.60 (− 1.00, − 0.21)	0.003	0.15 (− 0.22, 0.52)	0.421
SUA per 20-unit increase	608(100)	− 0.06 (− 0.11, − 0.02)	0.006	− 0.08 (− 0.13, − 0.04)	< 0.001	0.02 (− 0.03, 0.06)	0.396
MoCA							
Q1 (58.0–246.0)	151(24.8)	0		0		0	
Q2 (247.0–287.0)	150(24.7)	− 0.17 (− 0.69, 0.35)	0.519	− 0.13 (− 0.65, 0.39)	0.629	0.24 (− 0.19, 0.67)	0.268
Q3 (288.0–339.0)	155(25.5)	− 0.12 (− 0.64, 0.40)	0.647	− 0.02 (− 0.55, 0.50)	0.926	0.56 (0.12, 1.00)	0.012
Q4 (340.0–611.0)	152(25.0)	− 0.78 (− 1.30, − 0.26)	0.004	− 0.82 (− 1.38, − 0.26)	0.004	0.60 (0.10, 1.09)	0.018
*P* for trend		− 0.23 (− 0.39, − 0.06)	0.007	− 0.23 (− 0.41, − 0.05)	0.011	0.22 (0.06, 0.37)	0.007
Q1–Q3 (58.0–339.0)	356(75.0)	0		0		0	
Q4 (340.0–611.0)	152(25.0)	− 0.68 (− 1.10, − 0.26)	0.002	− 0.77 (− 1.21, − 0.32)	< 0.002	0.25 (− 0.13, 0.64)	0.198
SUA per 20-unit increase	608(100)	− 0.09 (− 0.14, − 0.04)	< 0.001	− 0.10 (− 0.15, − 0.05)	< 0.001	0.04 (− 0.01, 0.09)	0.111

Model 1: adjusted for age, gender, BMI, systolic blood pressure, education, diabetes and hypertension history, stroke subtypes, NIHSS scores, smoking and alcohol drinking status, serum total cholesterol, triglyceride, vitamin B12, folate, fasting glucose and UACR.

Model 2: Model 1 + eGFR.

Q1: quartile 1 (lowest quartile); Q2: quartile 2; Q3: quartile 3; Q4: quartile 4 (highest quartile).

*ß* standardized regression coefficient, *CI* confidence interval, *SUA* serum uric acid, *eGFR* estimated glomerular filtration rate, *UACR* urine albumin creatinine ratio, NIHSS National Institutes of Health Stroke Scale.

Table 3 shows the 1-month incidence of moderate-severe cognitive impairment across groups with different SUA levels. A total of 120 (19.7%) and 88 (14.5%) patients had moderate- severe cognitive impairment, as determined by the MoCA and MMSE, respectively. The results were similar to linear regression. In both crude and model 1, compared with the Q1 group, patients in Q4 had a higher risk of moderate-severe cognitive impairment at 1 month (adjusted OR = 2.36, 95% CI 1.09–5.12, *P* = 0.030 for MMSE; OR = 3.34, 95% CI 1.64–6.80, *P* = 0.001 for MoCA), whereas the direction of the relationship between SUA and incidence of moderate-severe cognitive impairment changed once eGFR was introduced into the model (OR = 0.64, 95% CI 0.25–1.62, *P* = 0.350 for MMSE; OR = 0.67, 95% CI 0.28–1.64, *P* = 0.384 for MoCA in Model 2). The outcomes were similar when SUA was analyzed as a dichotomous and continuous variable.

**Table 3:** Logistic regression analysis of the association between SUA levels and moderate to severe cognitive impairment.

SUA level (µmol/L)	No. of patients (%)	Crude		Model 1		Model 2	
		OR (95%CI)	*P*	OR (95%CI)	*P*	OR (95%CI)	*P*
MMSE							
Q1 (58.0–246.0)	16 (10.0)	1		1		1	
Q2 (247.0–287.0)	19 (12.0)	1.22 (0.60, 2.48)	0.576	1.20 (0.55, 2.59)	0.644	0.83 (0.37, 1.91)	0.669
Q3 (288.0–339.0)	23 (14.0)	1.47 (0.74, 2.91)	0.268	1.22 (0.56, 2.65)	0.615	0.69 (0.30, 1.61)	0.394
Q4 (340.0–611.0)	30 (19.7)	2.07 (1.08, 3.99)	0.029	2.36 (1.09, 5.12)	0.030	0.64 (0.25, 1.62)	0.350
*P* for trend		1.27 (1.04, 1.57)	0.022	1.31 (1.02, 1.68)	0.032	0.86 (0.64, 1.15)	0.313
Q1–Q3 (58.0–339.0)	58 (12.7)	1		1		1	
Q4 (340.0–611.0)	30 (19.7)	1.69 (1.04, 2.74)	0.035	2.04 (1.15, 3.61)	0.014	0.82 (0.41, 1.62)	0.563
SUA per 20-unit increase	88(14.5)	1.08 (1.02, 1.14)	0.013	1.10 (1.02, 1.18)	0.009	0.97 (0.90, 1.06)	0.531
MoCA							
Q1 (58.0–246.0)	20 (13.2)	1		1		1	
Q2 (247.0–287.0)	28 (18.7)	1.50 (0.80, 2.81)	0.201	1.58 (0.80, 3.15)	0.189	0.95 (0.44, 2.07)	0.900
Q3 (288.0–339.0)	30 (19.4)	1.57 (0.85, 2.91)	0.151	1.46 (0.72, 2.95)	0.293	0.66 (0.29, 1.50)	0.323
Q4 (340.0–611.0)	42 (27.6)	2.50 (1.39, 4.51)	0.002	3.34 (1.64, 6.80)	0.001	0.67 (0.28, 1.64)	0.384
*P* for trend		1.33 (1.10, 1.59)	0.003	1.43 (1.15, 1.79)	0.002	0.85 (0.64, 1.13)	0.265
Q1–Q3 (58.0–339.0)	78(17.1)	1		1		1	
Q4 (340.0–611.0)	42(27.6)	1.85 (1.20, 2.85)	0.005	2.41 (1.44, 4.03)	0.001	0.83 (0.43, 1.60)	0.573
SUA per 20-unit increase	120(19.7)	1.09 (1.04, 1.15)	< 0.001	1.14 (1.07, 1.21)	< 0.001	0.98 (0.91, 1.06)	0.630

Model 1: adjusted for age, gender, BMI, systolic blood pressure, education, diabetes and hypertension history, stroke subtypes, NIHSS scores, smoking and alcohol drinking status, serum total cholesterol, triglyceride, vitamin B12, folate, fasting glucose and UACR.

Model 2: Model 1 + eGFR.

Q1: quartile 1 (lowest quartile); Q2: quartile 2; Q3: quartile 3; Q4: quartile 4 (highest quartile).

*SUA* serum uric acid, *OR* odds ratio, *CI* confidence interval, *eGFR* estimated glomerular filtration rate, *UACR* urine albumin creatinine ratio, NIHSS National Institutes of Health Stroke Scale.

### Stratified analyses by potential effect modifiers

Stratified analyses were performed to further assess the relationship of SUA (the fourth quartile vs. the first three-quarters; Table 4) with cognitive performance in various subgroups. A fully adjusted stronger negative association between SUA and cognitive performance was found in those with lower eGFR with significant eGFR interaction for MMSE (*p*-interaction = 0.016) and MoCA (*p*-interaction = 0.005). None of the other variables, including gender, age, systolic blood pressure, NIHSS score, stroke subtypes, or blood glucose significantly modified the association between SUA and cognitive performance.

**Table 4:** Multivariable adjusted* linear regression of MMSE/MoCA scores with SUA within subgroups.

Subgroups	MMSE		*P* for interaction	MoCA		*P* for interaction
	Q1-Q3	Q4		Q1-Q3	Q4	
	ß (95%CI) *P*			ß (95%CI) *P*		
Gender			0.292			0.485
Male	0	0.06 (-0.38, 0.51)0.779		0	0.21 (-0.25, 0.66) 0.370	
Female	0	0.48 (-0.22, 1.19) 0.181		0	0.50 (-0.25, 1.25) 0.195	
Age dichotomous			0.160			0.594
Lower	0	-0.07 (-0.61, 0.48) 0.813		0	0.14 (-0.46, 0.74) 0.642	
Higher	0	0.45 (-0.08, 0.98) 0.099		0	0.35 (-0.18, 0.87) 0.198	
Systolic blood pressrue dichotomous			0.779			0.880
Lower	0	0.11 (-0.43, 0.66)0.682		0	0.20 (-0.36, 0.77) 0.481	
Higher	0	0.22 (-0.32, 0.75) 0.424		0	0.26 (-0.29, 0.82) 0.355	
NIHSS score dichtomous			0.867			0.393
Lower	0	0.13 (-0.44, 0.71)0.647		0	0.04 (-0.56, 0.63) 0.906	
Higher	0	0.07 (-0.43, 0.57) 0.778		0	0.37 (-0.15, 0.89) 0.168	
Stroke subtypes			0.548			0.997
Large artery	0	0.11 (-0.47, 0.68) 0.721		0	0.27 (-0.32, 0.86) 0.372	
Small vessel occlusion	0	0.33 (-0.17, 0.83) 0.202		0	0.27 (-0.27, 0.81) 0.331	
eGFR dichotomous			0.016			0.005
Lower	0	-0.65 (-1.20, -0.11) 0.020		0	-0.90 (-1.50, -0.29) 0.004	
Higher	0	0.02 (-0.60, 0.64) 0.946		0	0.12 (-0.55, 0.80) 0.724	
Blood glucose dichotomous			0.695			0.981
Lower	0	0.27 (-0.23, 0.76) 0.298		0	0.36 (-0.18, 0.89) 0.193	
Higher	0	0.10 (-0.46, 0.67) 0.718		0	0.25 (-0.32, 0.82) 0.391	

*SUA* serum uric acid, *ß* standardized regression coefficient, *CI* confidence interval, *eGFR* estimated glomerular filtration rate, *UACR* urine albumin creatinine ratio, *NIHSS* National Institutes of Health Stroke Scale.

*Adjusted, if not stratified, age, gender, BMI, systolic blood pressure, education, diabetes and hypertension history, stroke subtypes, NIHSS scores, smoking and alcohol drinking status, serum total cholesterol, triglyceride, vitamin B12, folate, fasting glucose, UACR and eGFR.

## Discussion

This study found a negative correlation between SUA level and cognitive performance in older adults with ischemic stroke and low eGFR in China; SUA increases were significantly positively associated with elevated risk of moderate-severe cognitive impairment and that severity of renal function influenced this relationship. This effect was present even after controlling for variables known to be associated with both cognition and elevated SUA. This partly supports the finding that, in CKD, the presence of elevated uric acid is associated with worse cognitive function^17^.

Of note, there are some contradictory data about SUA and cognitive dysfunction. Some studies reported that a higher level of SUA was associated with poorer cognitive performance^8^, while others suggested a beneficial effect^7^, and a possible effect of gender^16,38^. A prospective study in patients with ischemic stroke and TIA in China found a U-shaped association between SUA and PSCI in males but not in females^15^. Our study found that the relationship between SUA and cognitive function attenuated once eGFR was introduced into the model, in part confirming the association between eGFR, SUA and cognitive function. SUA may be a less informative risk factor of cognitive ability than eGFR in ischemic stroke patients.

The precise pathophysiologic mechanisms that underlie the link between the variations in uric acid, eGFR, and cognitive dysfunction after stroke are not known and require further elucidation. The relationship between eGFR and SUA levels was documented by numerous studies^21,39^. Hyperuricemia may potentiate the effects of angiotensin II to induce renal vasoconstriction, which could be mediated by its effect to upregulate angiotensin type 1 receptors on vascular smooth muscle cells, leading to arteriopathy, hypertension and kidney dysfunction^40^. In addition, it needs to be stressed that a low GFR value is a well-known risk factor for cognitive dysfunction^41^. A possibility may be that coexistence of lower eGFR and higher uric acid may also contribute to the development of vascular cognitive impairment by producing non-crystal associated renal and cerebral vascular injury, which underlies the development of moderate-severe vascular cognitive impairment. Coexistence of hyperuricemia and renal insufficiency may be a risk factor of PSCI. It remains unclear what the additional detrimental effect of these markers is on brain function. More research is needed to elucidate this issue.

The strengths of this study include its evaluation of cognitive function with both MoCA and MMSE, thus providing clinical accuracy to the analyses. In addition, we were able to control for a number of potential confounders while assessing the association, including demographic and clinical indicators.

There were several limitations to the design of our study. First, the SUA level was measured once, which ignored possible intra-individual fluctuations. Another consideration was that cognitive assessment was performed one month after hospital discharge, and the incidence of PSCI is highest three months after stroke^42^. Although there were no patients with dementia at baseline, since we did not take baseline cognitive score, we could not assess changes in cognitive level. Further long-term follow-up is still needed. Finally, we did not analyze impairment in specific cognitive domains, only compared overall cognitive scores.

## Conclusion

In summary, our findings suggest that in patients with ischemic stroke, SUA showed an inverse association with cognitive function in those with lower eGFR. We conclude that current findings support the hypothesis that severity of renal function may modify the relationship between SUA and cognitive performance. The findings have clinical implications. Patients with renal insufficiency should pay attention to uric acid level and actively control hyperuricemia to prevent cognitive dysfunction, or delay disease progression. Further studies are warranted to confirm whether uric acid lowering therapy in patients with renal dysfunction is a possible new determinant for cognitive impairment.

## Data Availability

The data that support the findings of this study are available from the corresponding author upon reasonable request.
